# Citizen journalism reduces the credibility deficit of authoritarian government in risk communication amid COVID-19 outbreaks

**DOI:** 10.1371/journal.pone.0260961

**Published:** 2021-12-08

**Authors:** Greg Chih-Hsin Sheen, Hans H. Tung, Wen-Chin Wu

**Affiliations:** 1 Department of Political Science, National Cheng Kung University, Tainan, Taiwan; 2 Department of Political Science, National Taiwan University, Taipei, Taiwan; 3 Center for Research in Econometric Theory and Applications, National Taiwan University, Taipei, Taiwan; 4 Institute of Political Science, Academia Sinica, Taipei, Taiwan; Universidade de Brasilia, BRAZIL

## Abstract

During the outbreak of an epidemic, the success in risk communications to make the public comply with disease preventive measures depends on the public’s trust in the government. In this study, we aim to understand how media audiences update their trust in the government during the COVID-19 outbreak depending on the information they received. We conducted an online survey experiment in February 2020 in Hong Kong (n = 1,016) in which respondents were randomly provided with a government press release and an endorsement either from an official or a non-official source. This study shows that the information from a non-official source enhances the credibility of official government messages. Our findings imply that dictators can actually “borrow credibility” from their citizen journalists and even nondemocratic leaders can make themselves more trustworthy to potential dissenters through citizen journalism. Allowing information flow from non-official sources can be a practical measure for governments to address the problem of a credibility deficit during a pandemic.

## Introduction

Can the trust in an authoritarian government be enhanced by endorsements from an independent source? The extant literature has documented various kinds of endorsement effects. In a democratic context, the endorsements from less biased newspapers win candidates more support from voters [1], and those made by celebrities also make them look more viable in electoral competition [2]. In a far more extreme scenario of civil wars, the outgroup leader endorsements for a peace settlement are found to work adversely on its public support [3]. Yet little is known if such an effect also exists in an authoritarian context. A related study [4] shows experimentally that the Chinese government can only rebut rumors and regain people’s trust in it when the rebuttals are endorsed by the public figures widely perceived to be independent. This study tries to enrich this literature on political endorsements under dictatorships by taking advantage of a narrow window of opportunity to conduct a survey experiment in Hong Kong (henceforth HK) during China’s COVID-19 outbreak. The timeliness of this research makes its findings less artificial since the informational environment and treatments administered in the experiments were real and vital to respondents’ lives amid the pandemic. Moreover, coupled with [4] in which the focus is more on how an authoritarian government *reactively* defends itself against rumors, this study further expands our understanding of the endorsements under dictatorships by analyzing how it can *proactively* persuade citizens of the credibility of its policies.

Specifically, our survey experiment finds that an authoritarian leader can actually “borrow credibility” from citizen journalist endorsements during a public health incident. Moreover, our findings also reveal that the effect is especially salient on those critical citizens who used to participate in HK’s 2019 Anti-Extradition Protest and favor media freedom in China. This implies that even nondemocratic leaders can make themselves more trustworthy to potential dissenters through citizen journalism.

In the current international endeavor for guarding global health against COVID-19, the role played by political systems has been placed under the spotlight. As the March 7 *Lancet* editorial [5] points out, China’s special “command-and-control political economy” in fighting the virus is absent in other nations, among which many are nondemocratic. And yet, their weaker authoritarian governments still will necessarily face the challenge during the global contagion. In other words, with less power and coercive apparatus than their Chinese counterpart, these governments’ ability to conduct risk communications in shaping the risk perceptions in their societies and instructing their citizens to adopt certain preventive measures such as social distancing and self-isolation becomes imperative during the current pandemic outbreak [6].

The success in risk communications to make the public comply with disease preventive measures has been found to be positively correlated with the trust in government [7–9]. More specifically, when it comes to the information distributed by the government, such trust often hinges on the credibility of its sources [10–12]. This is especially so when the information receivers, i.e. the public, do not possess adequate expertise in understanding technical details of the diseases, e.g., infection statistics and the effectiveness of vaccination [13]. However, an authoritarian government’s institutional incentive and capacity to manipulate information often make the public have little trust in its policy announcements and propaganda, even when it sincerely wants to open up and share truthful information with its citizens. For example, while some global health officials praised China’s authoritarian system for its prompt and forceful response to lock down the infected cities, the death of one of the eight arrested whistle-blowers in Wuhan, Dr. Li Wenliang, nonetheless reminded people that the outbreak could have been prevented if their voices were not censored by the local authority at the early stage when there were only a few local infections. Moreover, in addition to the problem in the internal transmission of information, an authoritarian government might also find its fragility in making its policies credible to the society it governs. The official notice of Wuhan’s lockdown on January 23 left an eight-hour window for people to decide whether they would leave or stay. According to Wuhan’s mayor, roughly 5 million Wuhan residents escaped from the city before it was actually enforced [14]. Furthermore, immediately after the lockdown, a second notice was issued to guarantee the sufficient supply of daily necessities and medical resources within the city. Nonetheless, it did not stop people from hoarding and trying their best to leave the city [15]. These incidents showed that the lack of the liberty of expression to health workers and media outlets might not only make an authoritarian government lose the opportunity to contain the virus ex ante, but also weaken its policy to stop its spread ex post.

Such a credibility deficit, therefore, presents a grave challenge to the ongoing global efforts in containing the coronavirus in countries with nondemocratic political systems. In fact, many experts have suggested that granting liberty of expression to citizens and journalists in authoritarian systems would be a solution [16]. In particular, since the public trust in the conventional media, which are usually viewed as government propaganda mouthpieces, tends to be low in authoritarian countries [17], a feasible solution to the authoritarian credibility deficit during a public health exigency should lie somewhere else: citizen journalism as an independent source.

There are three critical reasons for why citizen journalism is a good instrument for studying the endorsement effect under dictatorships. First of all, even in an authoritarian context where some kind of censorship is often imposed, citizen journalism has been found to liberate ordinary people from the government propaganda through providing alternative information [18, 19]. Second, it is also found that, unlike professional reporters, citizen journalists are less dependent on official and organizational sources for their reports [20], and therefore enjoy a higher level of independence from the government’s information manipulation. Finally, the susceptibility of citizen journalism to the government censorship (e.g., “frictions”) in China [21] might also raise its perceived independence among citizens. The perception may well arise from the simple fact that there is no reason for the government to censor citizen journalists’ reports if they are closely aligned with the official lines or citizen journalists can be co-opted. Based on these theoretical accounts, we hypothesize that citizen journalism endorsements can reduce the credibility deficit in public health governance and facilitate risk communications in authoritarian countries.

We conducted an online survey experiment (n = 1,016) in HK between February 13 and 17, 2020. It was right after the Chinese government confirmed more than 15,000 cases on February 12 alone due to a change in the diagnostic criteria. The survey questionnaire consists of four sections: Pre-experiment questions, Experiment 1, Experiment 2, and Post-experiment questions (Please see Supplemental Information H in S1 File for our complete questionnaire.). Experiments 1 and 2 were randomly ordered. In the pre-treatment section, we asked respondents questions such as their disease prevention knowledge and behavior, views on media freedom, participation in the 2019 HK anti-extradition protests, and knowledge about Chen Qiushi (henceforth CQ), a lawyer-turned-citizen reporter from mainland China. He was known by many HK citizens for his Youtube clips sympathizing with the anti-extradition protests. Among those who have heard of him, some considered him an independent and trustworthy journalist, while others viewed him as an agent of the Communist Party for manipulating HK’s public opinion in its favor. We leveraged this variation for checking if our manipulation worked. To avoid respondent fatigue, we left questions about demographic details and other information unaffected by the treatments in the post-treatment section.

Experiment 1 was designed to test the causal effect of the citizen journalism endorsement on the government news credibility. Subjects were first provided with a real press release about Wuhan’s ample supply of life essentials issued by China’s Ministry of Commerce (henceforth MoC). Then they were randomly presented with a MoC-endorsing montage screenshot taken from an online video report made either by the People’s Daily Online (henceforth PD) or CQ right after Wuhan’s lockdown. PD’s Online video (published on January 26, 2019, on v.eople.cn) is available at http://tiny.cc/fgxkkz and the screenshots were taken at the 0:33 and the 0:43 marks of the video. There was also a brief preamble introducing them: “Right the next day (January 26) after the Ministry of Commerce issued the press release, [a reporter from China’s official media outlet, People’s Daily Online,] visited a supermarket in Wuhan to do a live stream report on the shopping activities there. Here is a picture from the visit:.” The caption in the photo reads: “A People’s Daily Online journalist is visiting a supermarket in Wuhan: There is a sufficient supply of goods; Citizens are buying things in an orderly fashion.” (the 0:33 mark). The bracketed phrase indicated the information source (i.e., an official media outlet) to the respondents in this group. Alternatively, Chen Qiushi’s video (published on January 26, 2019, on Youtube) is available at http://youtu.be/KNLlYwTnY3E and the screenshots were taken at the 0:30 and the 1:42 marks of the video. As in the PD group, the same preamble except for the information source was also shown to introduce them: “Right the next day (January 26) after the Ministry of Commerce issued the press release, [a citizen journalist, Chen Qiushi,] visited a supermarket in Wuhan to do a live stream report on the shopping activities there. Here is a picture from the visit:.” The captions in the photo read “Other places are basically empty. All people are pretty much gathering here in the section for vegetables, fruits, and meats.” (the 0:30 mark) and “Hmm, there is also tons of rice.” (the 1:42 mark). In contrast, the bracketed phrase for this group indicated a non-official information source.

While both displayed a similar image of sufficient food for sale at a local supermarket in Wuhan, they differed starkly in the source —an official media outlet versus a citizen journalist. What should be noted here is that, owing to concerns over copyrights and personality rights of those who appeared in the screenshots, the screenshots are not shown in the published version of the paper, but available upon request.

All the information presented in this experiment is genuine. Before the experiment, we verified that both screenshots echoed MoC’s press release and were comparable to each other by consulting our colleagues. During the design stage of the survey, we shared the draft of the questionnaire to our colleagues at the following institutions: Academia Sinica, London School of Economics, National Taiwan University, New York University Abu Dhabi, the University at Buffalo, and the University of Washington. Overall we consulted 9 colleagues and asked them to comment on, in particular, whether the screenshots we used for both treatment and control groups were comparable to each other. All of them agreed with us about the comparability. After showing the screenshots to respondents, we asked them to rate the credibility of MoC’s press release and that of either PD’s or CQ’s report. We hypothesized that, because PD was a government mouthpiece and CQ was a non-official source viewed by many as independent, the rating should be higher when the press release was endorsed by CQ than by PD. Experiment 2 was designed to investigate whether mentioning an expert’s government-sponsored title would make his/her opinion more or less credible. To save space, we report its empirical results in (S2 Fig).

## Materials and methods

We conducted an online survey experiment in HK between February 13 and 17, 2020. The experiment design obtained Institutional Review Board approval at New York University Abu Dhabi (HRPP-2020–15) on February 3, 2020. With the gender and the age quotas derived from the 2018 Population and Household Statistics published by the HK government, a total of 1,016 respondents (aged 18 to 80) were recruited through the proportional quota sampling by the Rakuten Insight Global, an international survey company. What is worth noting here is that a common feature of online surveys is over-representation of highly educated respondents. Our respondents also reported higher educational levels (approximately 65% have a university or above educational level) than the general population (approximately 33%). However, this is less of an issue for our study since the main instrument of our research, citizen journalism, replies heavily on online publications [20] and its influence will certainly concentrate more on the population with a higher level of education.

Moreover, a power analysis was conducted to decide our sample size. Our goal was to obtain 0.90 power to detect an effect size of 0.3 at the 0.001 alpha error probability. This analysis yielded a sample size of 930 with 465 participants in each group, and we recruited slightly more participants than the suggested amount. Apart from the demographic questions, to minimize the possibility that inattentive responses may impair data quality, choices of all the other ones were randomized. Overall, there are 47 questions in the questionnaire. The ex ante estimated completion time is 10 minutes. The average completion time of our data is 12 minutes. Please see S1 File for further details about our participant recruitment and human subjects protection. We chose HK to conduct this experiment since it’s a part of China that still enjoyed partial media freedom, and its citizens were more experienced in detecting government propaganda. In other words, if exposing them to our informational treatment did induce an endorsement effect on a government announcement, then it should be easier to observe such an effect among other less sophisticated respondents. Moreover, since HK citizens were less likely to have a very concrete idea about Wuhan’s lockdown when our experiment was administered, they would also be more responsive to our experimental manipulations.

### Vignettes and questions of Experiment 1

The government press release was presented as follows:

The Novel Coronavirus Pneumonia is spreading. At the moment of announcing the lockdown of the city of Wuhan, the Ministry of Commerce, People’s Republic of China issued a press release titled “The commerce department of Hubei is making all efforts to ensure the sufficient supplies of life essentials,” including the following statement: “On January 24, the city of Wuhan is supplied with sufficient life essentials, and the price of vegetables also decreased. The commerce department of Hubei is making all efforts to ensure the sufficient supplies of life essentials. First, the government will supply the essentials for Lunar New Year celebrations at markets. 1.55 million kilograms of eggs, 5 million kilograms of vegetables, 1 million kilograms of fish, 0.2 million kilograms of beef and 6000 heads of pigs will be distributed across 300 stations. Starting January 21, 0.5 million kilograms of frozen pork have also been distributed in cooperation with the Development Department. Second, to ensure the normal operation of retailing businesses in Wuhan, we ask all supermarkets, pharmacies, and gas stations to continue their operations during the period of Lunar New Year and increase their stocks. Third, all vegetables and meats will be shipped into Wuhan through the 24-hour Green Lane, to ensure smooth transportation. Fourth, we will continuously inspect the market supplies of life essentials and address any issues immediately.”

The Screenshots used in this experiment were then shown to respondents and the sources were:

- Press release from the Ministry of Commerce of the People’s Republic of China (published on January 25, 2019) http://www.mofcom.gov.cn/article/jiguanzx/202001/20200102932965.shtml
- Chen Qiushi’s video (published on January 26, 2019, on Youtube) http://youtu.be/KNLlYwTnY3E
- People’s Daily Online video (published on January 26, 2019, on v.eople.cn) http://tiny.cc/fgxkkz

The survey questions included:

- Do you believe in the Ministry of Commerce’s press release? Rate it on a scale from 0 to 100. A larger number indicates higher level of trust.
- Do you believe that the report by Chen Qiushi faithfully reflected the situation in Wuhan? Rate it on a scale from 0 to 100. A larger number indicates higher level of trust.
- Do you believe that the report by the People’s Daily Online faithfully reflected the situation in Wuhan? Rate it on a scale from 0 to 100. A larger number indicates higher level of trust.

### Hypothesis and vignettes of Experiment 2

Experiment 2 was designed to investigate whether an expert opinion with or without mentioning the expert’s government-related title had a different level of credibility. We studied this using a vignette quoted from a Hong Kong newspaper. In the news piece, the expert cited, Dr. Li Xingwang, was presented as “a member of the National Medical Expert Committee and Chief Expert at Clinical and Research Center of Infectious Diseases, Beijing Ditan Hospital.” We randomly provided the respondents with the vignette that included or excluded the statement that Dr. Li was a member of National Medical Expert Committee. Then we asked respondents to rate their trust in Dr. Li’s statement. Our aim was to investigate how Dr. Li’s opinion was considered as more or less credible depending on whether or not his government-related title was cited. Being included on the government expert committee, on the one hand, reflects that his expertise is highly regarded. On the other hand, however, it also means that he may be incentivized to align more closely to the government. Hence, his opinion may not be considered as purely neutral and science-based. Accordingly, we tested the following hypothesis:

The mean perceived credibility of Dr. Li’s opinion differs between the group of respondents who read the vignette with his government-related title and the group who read the vignette without the title.

Vignette:

Dr. Li Xingwang, [a member of the National Medical Expert Committee and] Chief Expert at Clinical and Research Center of Infectious Diseases, Beijing Ditan Hospital, suggests that, as the virus starts to spread nationwide, there are many infected cases all over the country. Some contagious patients may display no symptoms but are later tested positive. Some others will show no obvious fever but sometimes cough, and feel fatigue. “These kinds of patients are also contagious. However, as the virus is spread by droplets, and those with mild illness tend to show fewer cough symptoms… the transmission ability might not be that strong,” said Dr. Li.

The source of news is HK01 (a Hong-Kong-based online newspaper) http://tiny.cc/0mskkz, and we asked the following question:

Do you believe in the opinion of Dr. Li Xingwang, [a member of the National Medical Expert Committee and] Chief Expert at Clinical and Research Center of Infectious Diseases, Beijing Ditan Hospital? Rate it on a scale from 0 to 100. A larger number indicates higher level of trust.

## Results

We used two-tailed t-tests to perform randomization checks. As reported in S1 and S2 Tables in S1 File, the p-values of these t-tests indicate that the group differences are insignificant in our two experiments, except for the gender in Experiment 2. We however would like to note that although randomization checks are widely used for assessing whether a random assignment is conducted properly, it is still controversial if this practice can really fulfill its promise [22]. The average treatment effects of major interest include (1) the difference in the perceived credibility of the government press release between the group receiving the government-endorsing information from a state-owned media outlet and that receiving the similar information from a citizen reporter, and (2) the difference in the perceived credibility of a medical expert’s statement with or without his government-sponsored title mentioned. We also ran OLS regression and linear interaction models for estimating heterogeneous treatment effects.

**Fig 1:**
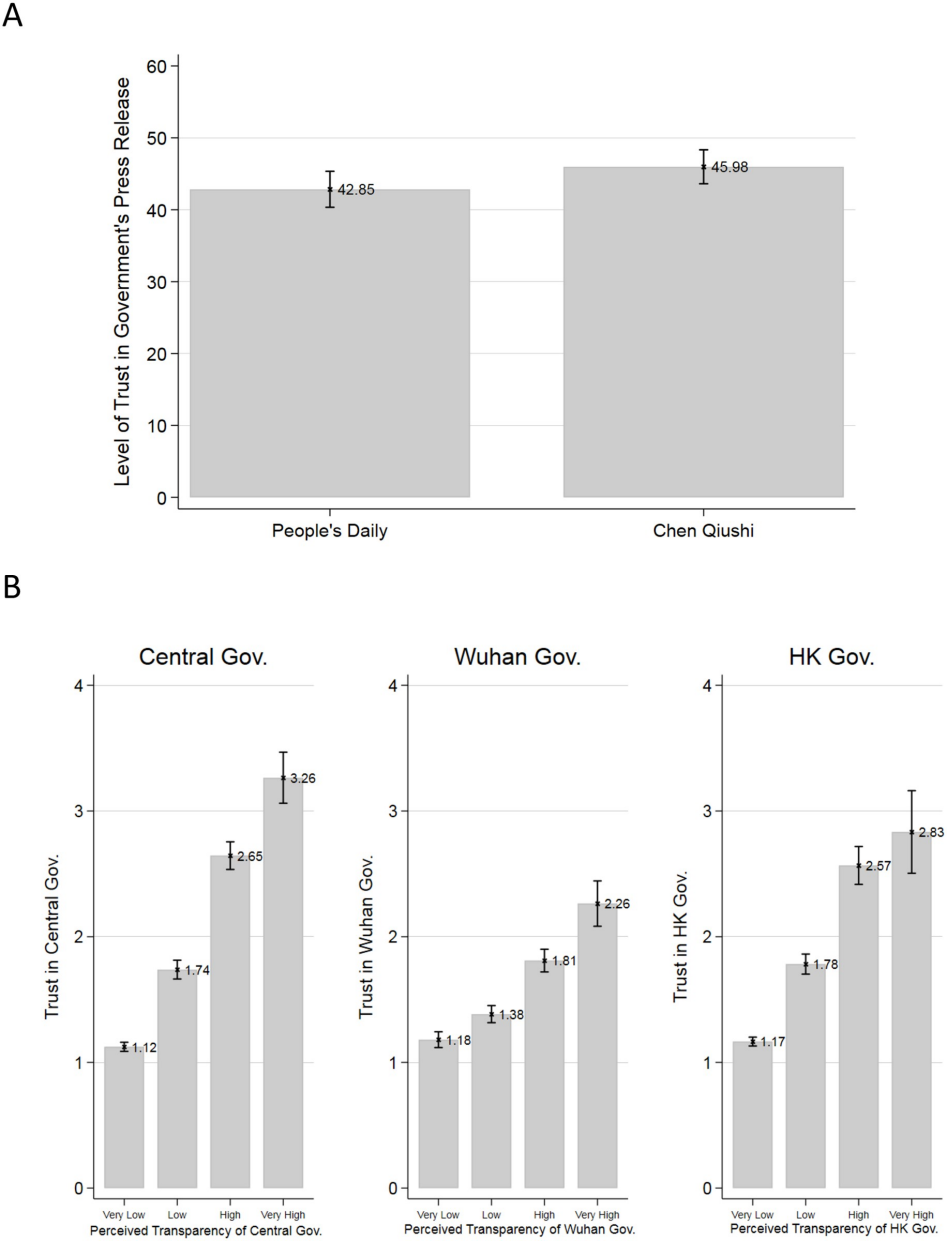
Endorsement effects of citizen journalism. Trust in government press release after reading news from different sources (A), and government transparency in disease information and trust in government (B).

We found that the exposure to independent citizen journalism increased respondents’ trust in the government press release. A two-tailed t-test shows that the respondents who read CQ’s report gave higher ratings of credibility to MoC’s press release than those who read the PD report (45.98 versus 42.85, the difference is 3.13, two-sided p-value = 0.07, see Fig 1A). More generally, we also found that, across all levels of the government, the general trust in them correlated positively with respondents’ perceived transparency of information concerning the outbreak (Fig 1B).

**Fig 2:**
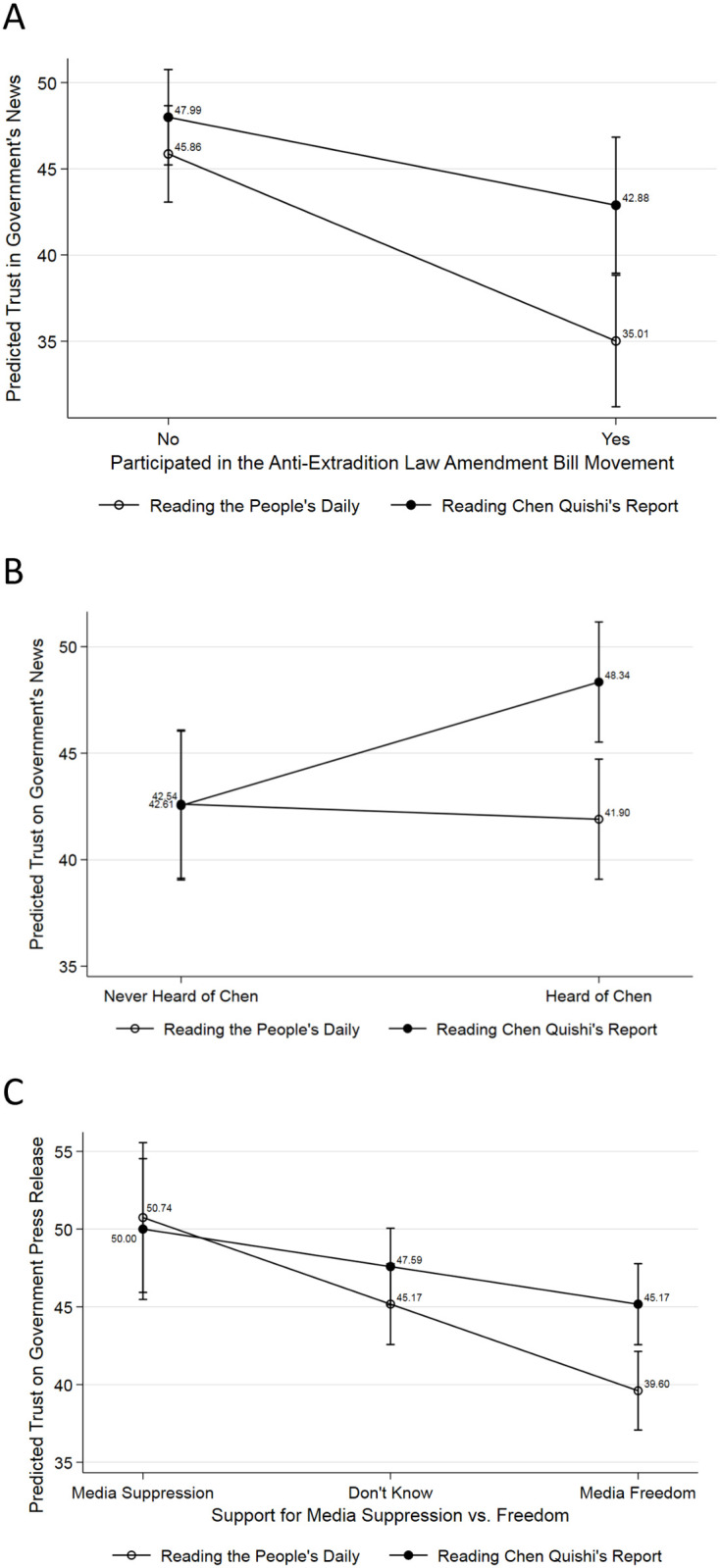
Causal heterogeneities. Social movement Participation and trust in government press release (A), previous awareness of CQ and trust in government press release (B), and support for media freedom, information sources, and trust in government press release (C).

To further explore the heterogeneous effects of independent citizen journalism on reducing the authoritarian credibility deficit, we estimated several linear regression models including respondents’ demographics and virus-related variables (see S3-S5 Tables in S1 File for details). There was substantial evidence showing that the identified causal effect was driven by the respondents who were more critical. First, tapping into HK’s recent critical event, the 2019 anti-extradition protests, we found CQ’s report induced a greater trust on MoC’s press release among protest participants than among non-participants (Fig 2A, 42.88 versus 35.01, the difference is 7.87, *p* = 0.003). Moreover, as the majority of protesters were relatively young, it’s therefore also worthwhile to investigate the causal heterogeneity across different age groups (18–80 in our sample). Just like the analysis on protest participation, we did find statistically significant and larger independent treatment effects among relatively younger (24–52) participants (S3 Fig). Please see section G in S1 File for details. Similarly, those who heard of CQ and favored media freedom in mainland China also showed a higher trust in the government press release after reading CQ’s report (Fig 2B, 48.34 versus 41.90, the difference is 6.44, *p* = 0.001; Fig 2C, 45.17 versus 39.60, the difference is 5.57, *p* = 0.002).

**Fig 3:**
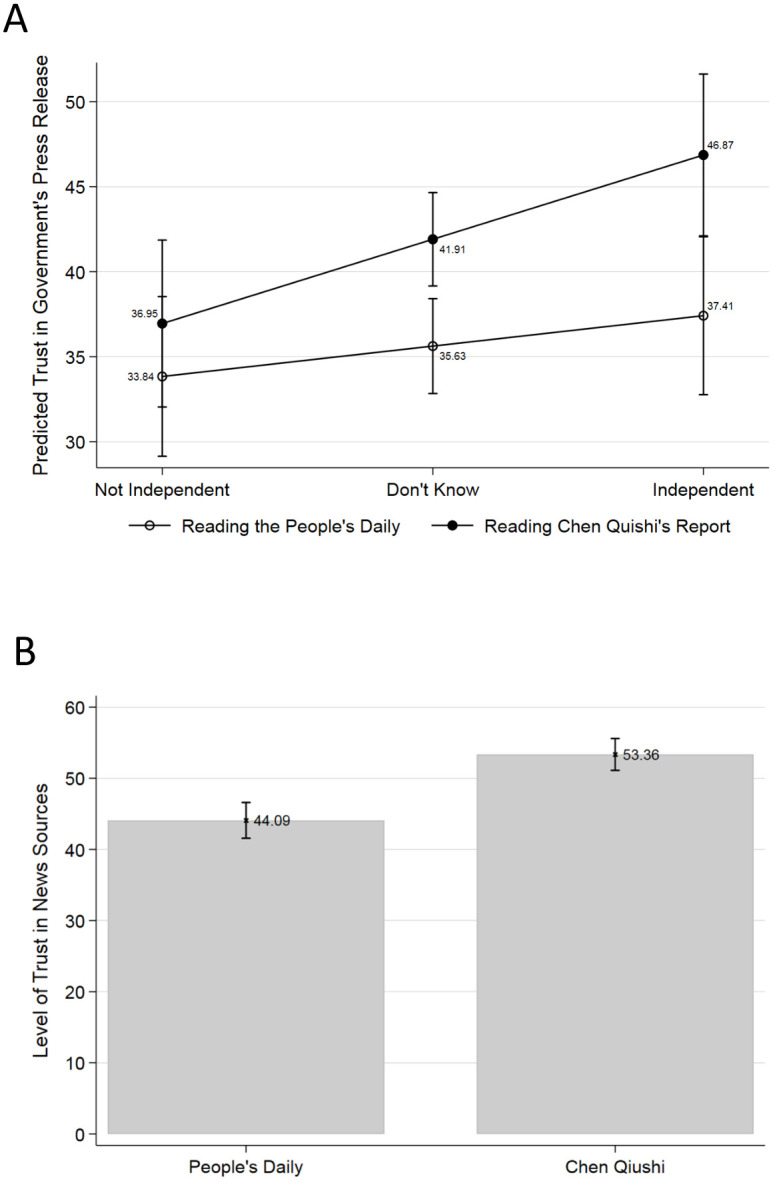
Manipulation checks. CQ’s independence and trust in government press release (A), and trust in different sources of information (B).

Finally, the manipulation checks are also consistent with our expectations. Respondents showed higher levels of trust in government press release as they perceived CQ to be an independent reporter (46.87, *p* < 0.001, see Fig 3A). Similarly, compared to the credibility of PD report, on average, respondents rated CQ’s report with a higher level of trust (44.09 versus 53.36, the difference is 9.27, *p* < 0.001, see Fig 3B).

## Discussion

Our study shows that, during a public health crisis, the credibility deficit of an authoritarian government can be substantially reduced by citizen journalism. Such an effect is especially salient among those citizens who are critical of the government. We draw four conclusions from our analysis.

First, as the number of newly confirmed cases has gradually flattened out in China and went up elsewhere, it has become the global priority to find a practical solution to reducing credibility deficit in risk communications in other authoritarian countries without China’s administrative capacity. The observed effects of citizen journalism from our experiment were moderate yet meaningful. What is worth mentioning here is that the effects of our informational treatments might be moderated by the fact that, given Hong Kong’s free internet and media environment, our respondents also had low-cost access to other information sources regarding Wuhan’s local conditions that might neutralize CQ’s reports. This informational decentralization can also help account for why the government-related title in our second experiment was unable to induce any difference in respondents’ trust in medical experts since they did not need to rely on government sources for information. This is especially worth noting since the effects were triggered simply by a brief visual treatment. More importantly, while the effects of risk communications tend to be dwarfed by those exerted by preventative measures themselves, they can still be quite substantial in absolute numbers on a population level [23, 24].

Second, the effect is also strengthened by the finding that, even in an authoritarian country, one will trust the government more when his/her perceived government transparency in handling the outbreak is higher (Fig 1B). The fact that the effect is significant across all levels of government suggests that citizen journalism can help an authoritarian government enhance its credibility in both central decision-making and local implementation of public health policies.

Third, besides showing the average causal effect of independent citizen journalism in an authoritarian context, our research also unpacks several mechanisms driving it. Actually, one might wonder if our results were mainly driven by the regime’s loyal supporters. If this is the case, lifting the restrictions on citizen journalism probably won’t help an authoritarian government much in reducing its credibility deficit since loyalists would support it anyway. Moreover, there have also been various studies showing how destabilizing public health hazards can be to authoritarian countries [25, 26], and a sudden removal of censorship might give potential rebels opportunities to mobilize and paralyze their governments’ ability to deal with such crises [27].

Our findings, by contrast, suggest otherwise and therefore should send a soothing message to authoritarian leaders who are facing the COVID-19 challenge. According to our analysis of causal heterogeneities, while the respondents who were participants of anti-extradition protests and supporters of media freedom in China had lower trust in the government press release to begin with, these potential dissidents however, upwardly adjusted their trust in it significantly more than their not-so-critical counterparts when they received the government-endorsing information from a non-official source. In other words, there can actually be a virtuous cycle between citizen journalism and government credibility under dictatorships.

Fourth, our results also have a profound implication for the state-society synergy in public health governance under authoritarian regimes. It has been well-established in the literature that social capital can not only help authoritarian governments provide more local public goods [28], but also promote better public health [29–31]. Our results offer yet another piece of evidence for how having a vibrant civil society where independent citizen journalism is able to thrive can reduce dictators’ credibility deficit during an epidemic outbreak.

Our study, however, has one major limitation. We only showed our respondents the kind of citizen reports that endorsed the government press release. We were constrained to identify one of CQ’s reports that could be juxtaposed symmetrically with a government press release related to the outbreak. Unfortunately, because CQ stopped twittering in early February, his January clip on the food supply at one of Wuhan’s supermarkets was the only one that qualified (What has to be emphasized here is that our experiment was administered after CQ disappeared from the public sight.). Does this affect CQ’s perceived credibility as an independent source and therefore undermine our argument? Fortunately, this issue is partially addressed by the effect of CQ’s independence we found (Fig 3A). Since the independence in citizen journalism implies that one’s reports won’t be always endorsing but also critical, counter-factually, this finding suggests that the endorsement effect is unlikely to disappear even when citizen journalists’ reports contradict the government’s announcements. In other words, even if we were only able to present CQ’s endorsement of the government policy, our respondents didn’t regard him as simply a mouthpiece.

In this race against the spread of the virus, our study shows that allowing some level of free flow of information can be a practical way for authoritarian governments that suffer from credibility deficit to improve public trust in their risk communications and induce people’s cooperative behavior in containing the contagion during the current pandemic outbreak.

## Concluding remarks

The effects of citizen journalism on non-democratic regimes’ credibility deficit in risk communication during public health crises are only partially understood. In general, the traditional model of risk communication stresses the importance of facilitating the transmission of information from authoritative/official sources. Citizen journalism is, therefore, viewed as a positive factor that can not only provide more information that is inaccessible to government sources, but also proactively engage those who are affected to shape their risk perceptions. In the authoritarian context where some kind of censorship is often imposed, citizen journalism has been found to liberate people from propaganda through providing alternative information and aggravate dictators’ credibility deficit. However, little is known if citizen journalism in a dictatorship can conversely work in tandem with its authoritarian government to enhance the effectiveness of risk communications during a public health crisis.

To the best of our knowledge, this study is the first to confirm the positive effect of citizen journalism on reducing the credibility deficit in public health governance in authoritarian countries. First of all, we took advantage of a narrow window of opportunity to conduct a survey experiment in Hong Kong during China’s COVID-19 outbreak. The timely research makes our findings less artificial and closer to reality. Second, compared to the previous study, we show that a citizen journalist’s report can enhance the credibility of dictatorships. Third, in addition to the average causal effect of citizen journalism on authoritarian credibility in risk communications during a public health crisis, our findings also reveal that the effect is especially salient on those critical citizens who used to participate in Hong Kong’s 2019 Anti-Extradition Protest and favor media freedom in China.

The study shows that authoritarian leaders can “borrow credibility” from citizen journalists during a public health incident. Granting citizens freedom of speech can make authoritarian regimes more, instead of less, credible. This implies that there can actually be a synergy between state and society in a dictatorship. Given our finding that critical citizens also tend to be more responsive to reports made by citizen journalists, it implies that even nondemocratic leaders can make themselves more trustworthy to potential dissenters through citizen journalism. Moreover, the kind of experimental manipulation our participants were exposed to was not simply about some generic information about COVID-19, but about an actual policy measure that might affect people’s behavior in real-life scenarios. This also renders our findings readily applicable to actual anti-virus campaigns in other authoritarian countries. Finally, based on our findings, we also open up a new avenue for future researchers to explore various ways in which authoritarian governments are able to reduce their credibility deficit. Especially, the conventional wisdom tends to focus on how international institutions can make them more trustworthy to foreign actors such as multinational companies and investors. Our study shows alternatively how they can also gain additional credibility by tapping into civil society groups for sources of independence. Future research therefore can explore other domestic actors and issues areas to enrich our understanding of “dictators’ credibility borrowing”.

## Supporting information

S1 Fig The informed consent page. (TIF)

S2 Fig Government title and credibility of a medical expert. (TIF)

S3 Fig Age and heterogeneous treatment effects. (TIF)

S1 File (ZIP)
