# Supplementary figures and images for: Citizen journalism reduces the credibility deficit of authoritarian government in risk communication amid COVID-19 outbreaks
Source: PLoS One. 2021 Dec 8;16(12):e0260961. doi: 10.1371/journal.pone.0260961 (PMC8654212; doi:10.1371/journal.pone.0260961)

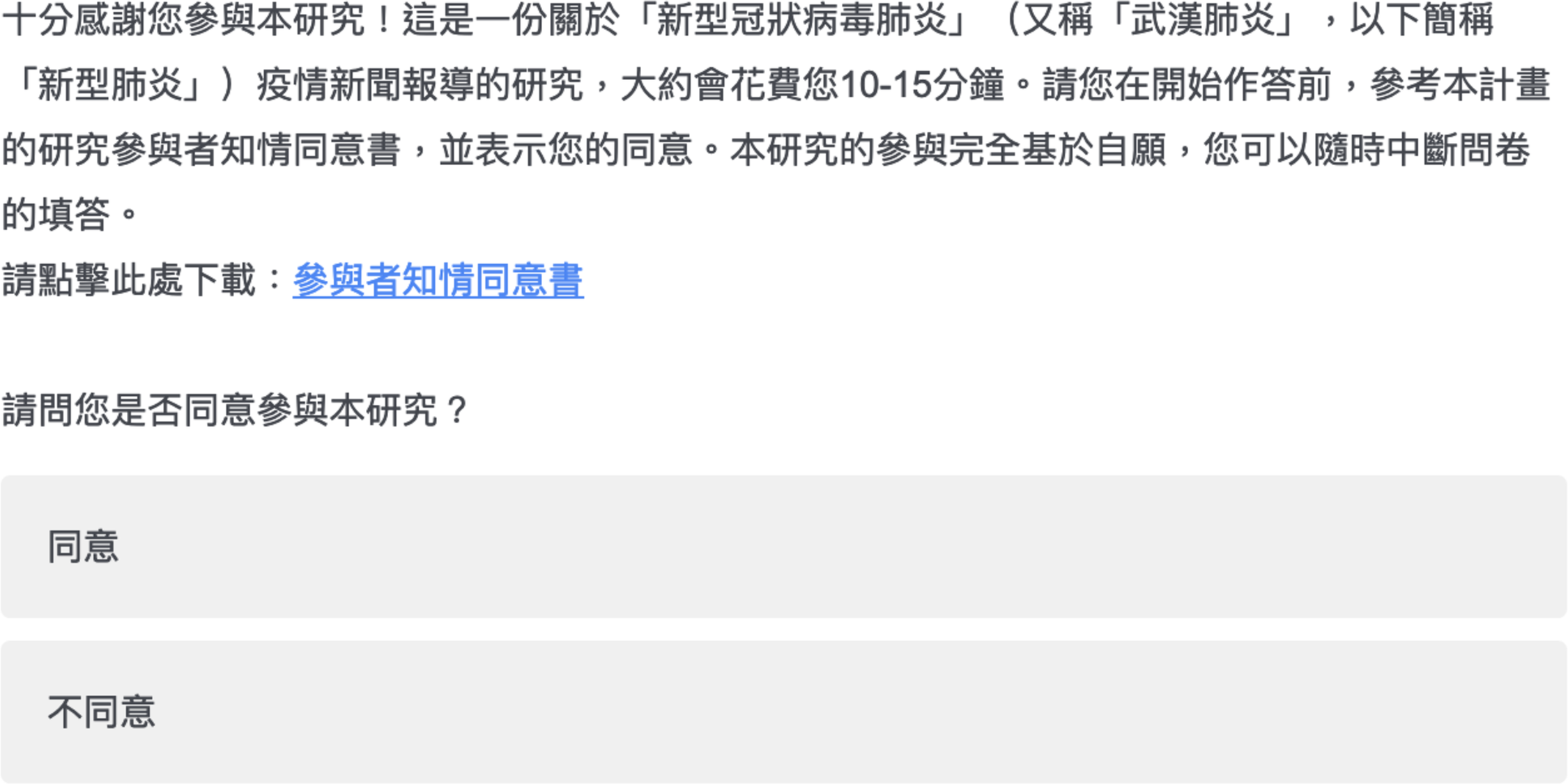

Supplement: S1 Fig — (TIF) [file pone.0260961.s001.tif]

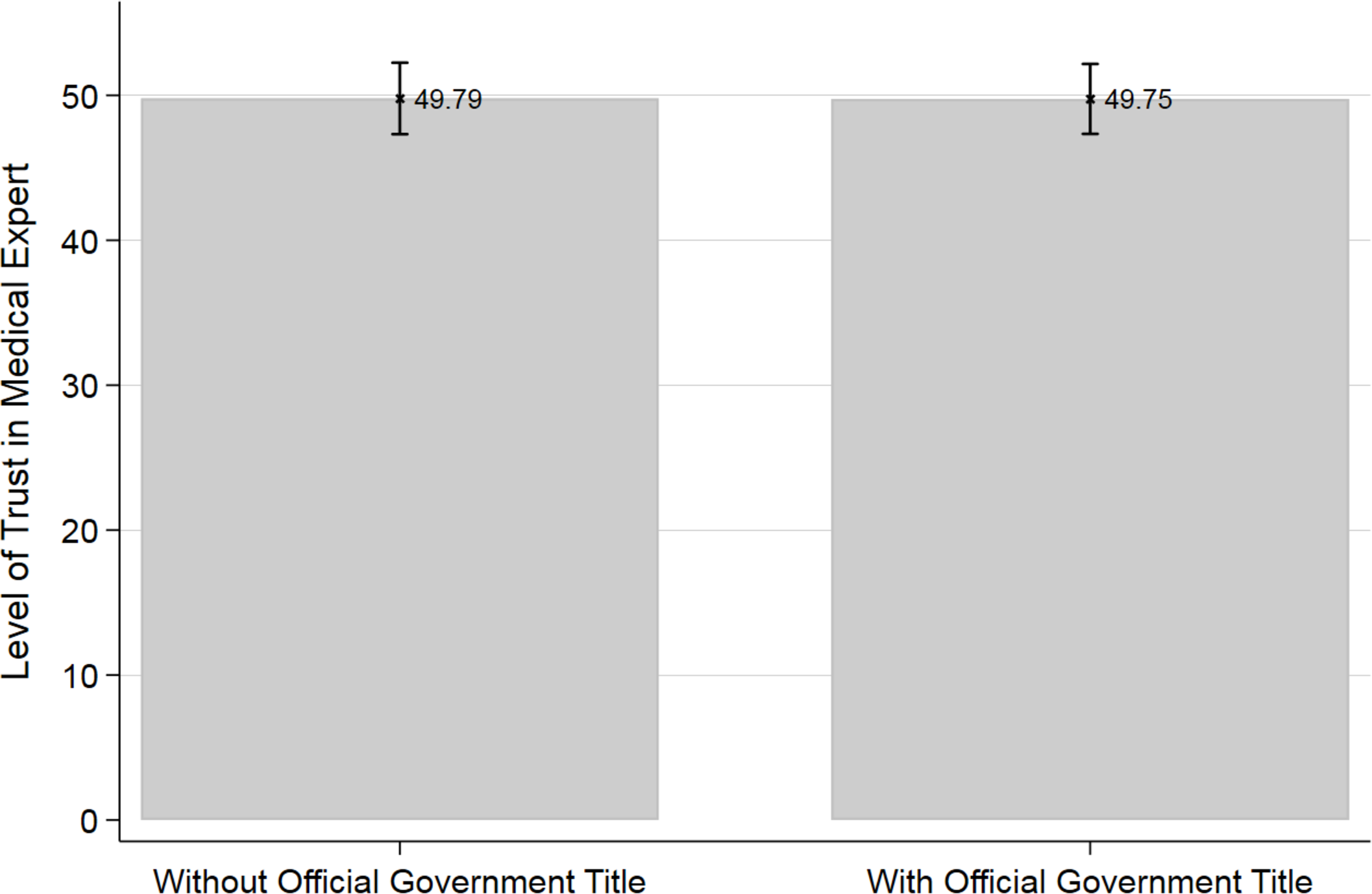

Supplement: S2 Fig — (TIF) [file pone.0260961.s002.tif]

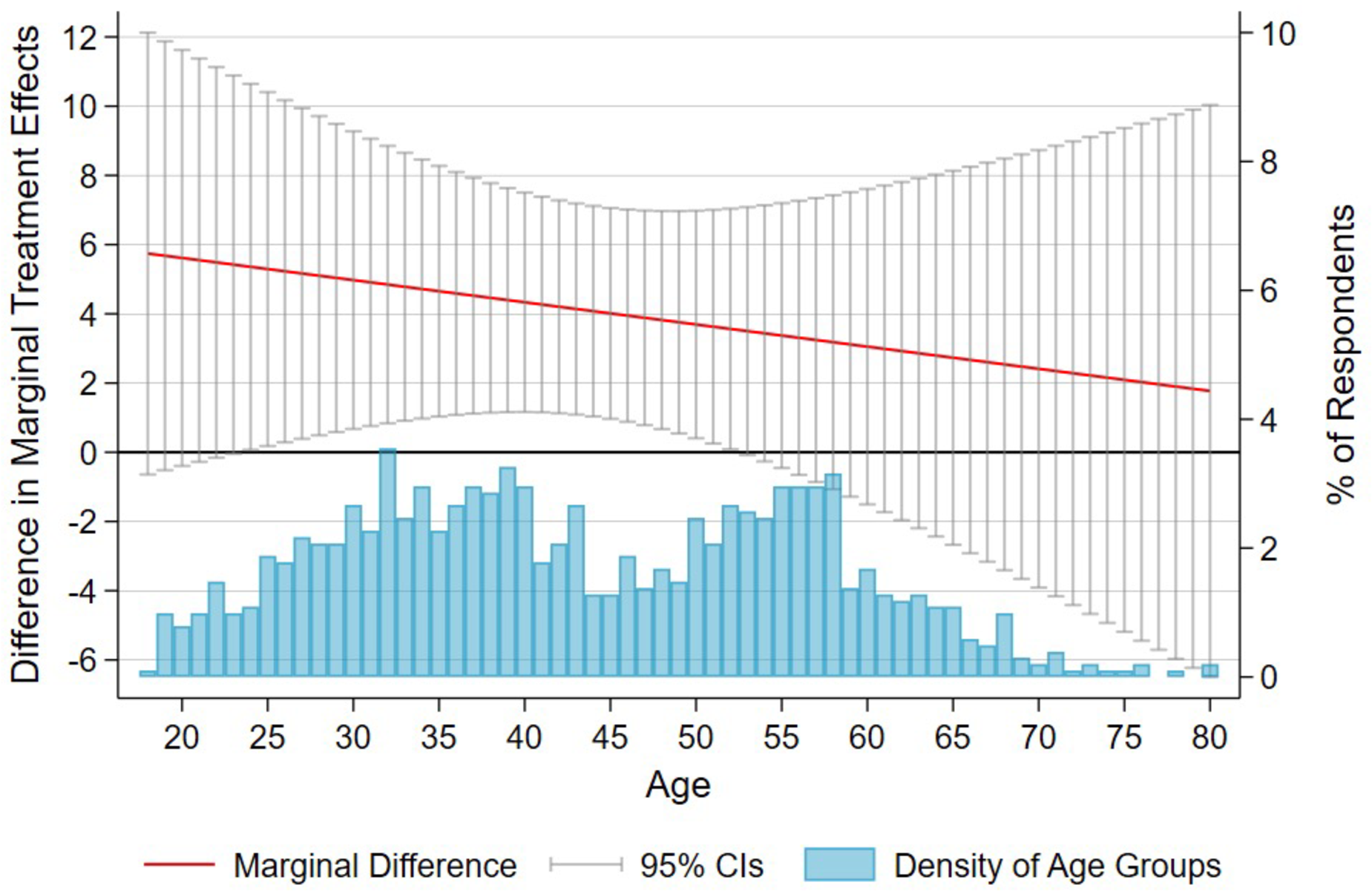

Supplement: S3 Fig — (TIF) [file pone.0260961.s003.tif]
