# Supplementary material for: Citizen journalism reduces the credibility deficit of authoritarian government in risk communication amid COVID-19 outbreaks
Source: PLoS One. 2021 Dec 8;16(12):e0260961. doi: 10.1371/journal.pone.0260961 (PMC8654212; doi:10.1371/journal.pone.0260961)
Supplement: S1 File — (ZIP) [file pone.0260961.s004.zip › Supporting Information_final.pdf]

Supporting Information for Citizen journalism reduces the credibility deficit of authoritarian government in risk communication amid COVID-19 outbreaks

Greg Chih-Hsin Sheen[1◑], Hans H. Tung[2,3*◑], Wen-Chin Wu[4◑],

1 Department of Political Science, National Cheng Kung University, Tainan, Taiwan
2 Department of Political Science, National Taiwan University, Taipei, Taiwan
3 Center for Research in Econometric Theory and Applications, National Taiwan University, Taipei, Taiwan
4 Institute of Political Science, Academia Sinica, Taipei, Taiwan

◑These authors contributed equally to this work.
¤Current Address: Department of Political Science, National Taiwan University, 1 Sec. 4 Roosevelt Rd., Taipei, 10617, Taiwan
* hanstung@ntu.edu.tw

Supplementary information A: Participant Recruitment and Human Subjects Protection

De-identification

The voluntary participants of our study were recruited from Rakuten Insight's (`https://insight.rakuten.com`) proprietary online panel in Hong Kong. Rakuten Insight, a leading global survey company, adopts a strict privacy policy on for their members (`https://insight.rakuten.com/privacypolicy/`) and adheres to the Binding Corporate Rules (`https://ec.europa.eu/info/law/law-topic/data-protection/international-dimension-data-protection/binding-corporate-rules-bcr_en`), a global privacy protection standard in data transfers. This provides the first layer of protection for our respondents' privacy. More critically, we only received de-identified responses from our participants. We neither have any identifying information about them, nor know how to trace collected responses to any specific individuals.

Informed Consent

At the very beginning of our survey, respondents were presented with the informed consent form and given an opportunity to show their consents. In other words, our participants knew perfectly they were about to take part in a research study, of which the information about the principal investigators, the design of the study, and their protection was fully provided. Moreover, this study involved no deception in the design. Figure S1 Fig presents the screenshot of the page (The English translation follows).

English Translation

Thank you very much for your participation! This is a study about the news reports on the "Novel Coronavirus Pneumonia" (aka the "Wuhan Pneumonia," henceforth "Novel Pneumonia" as a shorthand) outbreak. The survey will take you about 10-15 minutes to

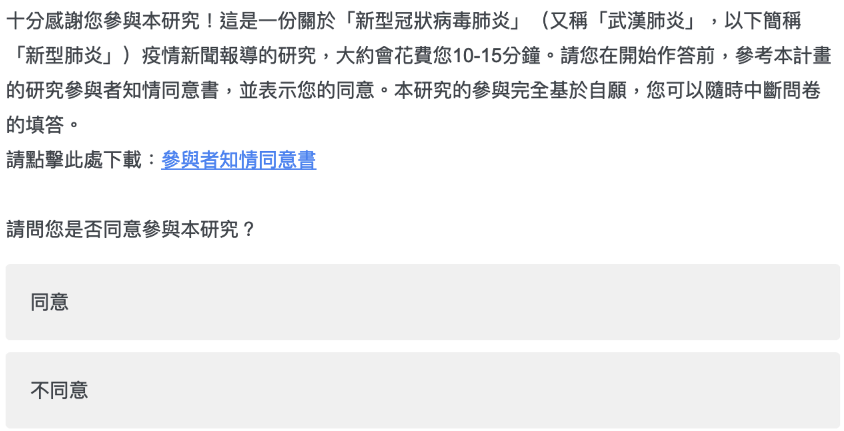
十分感謝您參與本研究！這是一份關於「新型冠狀病毒肺炎」（又稱「武漢肺炎」，以下簡稱「新型肺炎」）疫情新聞報導的研究，大約會花費您10-15分鐘。請您在開始作答前，參考本計畫的研究參與者知情同意書，並表示您的同意。本研究的參與完全基於自願，您可以隨時中斷問卷的填答。
請點擊此處下載：參與者知情同意書

請問您是否同意參與本研究？

同意

不同意

S1 Fig. The Informed Consent Page.

finish. Please read the following informed consent form carefully and give us your consent before you start. Participation in the survey is completely voluntary. You are free to withdraw from the study at any point.

Please click here for downloading the form: Informed Consent Form.

Do you consent to participate in this study?

- Yes
- No

Minimizing Political Risks

The survey was administered in mid-February this year (2020) when the Anti-Extradition Protests had gradually died down owing to the COVID-19 outbreak and the government's social distancing policy[1]. It was also way before the passage of the National Security Law by China's National People's Congress in June. In other words, this was a period when the political tensions in Hong Kong were relatively low and it was less risky for our respondents to take part in a study like ours then.

That said, there have been three steps taken to minimize the potential political risks for our respondents. First of all, as we explained above, Rakuten Insight adheres to the global standard in protecting the privacy of their members. More critically, while Rakuten Insight had access to the list of participants of our study, it didn't have access to their de-identified responses, which were recorded by Qualtrics, the online survey platform we used.

Second, to minimize the risks for our respondents, we also designed the question about the protest participation (See S3 Table Table) in such a way that it was vague (yet informative) enough for those who gave a positive answer to exonerate themselves. As a matter of fact, since mid-June, 2019, there had been a series Anti-Extradition Protests taking place in Hong Kong and not all of them were illegal and violent. In other words, saying yes to our question might well be just an indication that they joined one of the legal and peaceful demonstrations at the early stage of the Protests. Simply put, our protest participation question can't be the basis for an indictment.

[1]Virus puts Hong Kong protests on ice. Will they return? By Eileen Ng, February 11, 2020. Associated Press. Available at: `https://apnews.com/9425f9bc7dec69e05d7b76dde903e70d` [Accessed March 20, 2020].

Third, we have deleted the survey along with all the responses recorded by Qualtrircs. The downloaded data have also been encrypted and stored off-line only. In addition, the data can only be accessed with passwords by the three authors of the paper.

Fair Compensation

Finally, in terms of the compensations we made to the respondents, our rate was substantially higher than Hong Kong's Statutory Minimum Wage (SMW). According to Hong Kong government recent announcement (https://www.labour.gov.hk/eng/legislat/content5.htm), the current SMW rate is $4.8 USD ($37.5 Hong Kong Dollars) per hour. Our study, by contrast, paid our participants roughly $8.7 USD per hour ($2.175 USD for a 15-minute survey).

Supplementary information B: Results of Experiment 2

The second main outcome of interest is respondents' trust in a medical expert's explanations of the coronavirus. Respondents were asked to rate their trust in the explanations provided by a medical expert with and without the expert's government-related title included in the statement on a scale from 0 to 100, with a larger number indicative of a higher level of trust. We hypothesized that the credibility of medical experts with a government-related title would be lower than that of medical experts without the title. Yet, we found that our data did not support this hypothesis. As shown in S2 Fig Fig, the difference between the two groups is only 0.04 on a scale from 0 to 100 (95% CI -3.41-3.47). As a result, the result does not support our second hypothesis that the inclusion of government-related title would matter to an medical expert's statement about the disease.

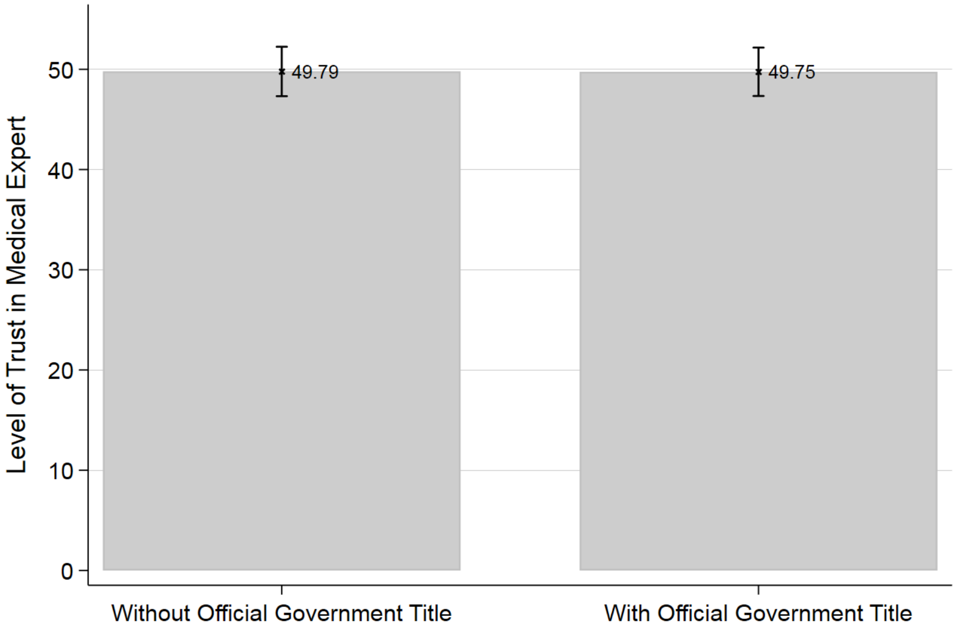

S2 Fig. Government title and credibility of a medical expert

Supplementary information C: Randomization Checks

S1 Table. Randomization checks: Experiment 1

Variable	Group People's Daily			Group Chen Qiushi			p-value
	N	Mean	SD	N	Mean	SD	
Age	489	44.043	13.232	495	42.962	13.127	0.199
Male	490	0.486	0.500	497	0.489	0.500	0.920
College	496	66.330	0.473	496	0.663	0.473	0.890
Subjective Social Class	471	2.270	0.905	483	2.308	0.829	0.489
Party ID	490			497			
Pro-establishment	61	0.125	0.330	68	0.137	0.344	0.566
Moderate	140	0.286	0.452	134	0.270	0.444	0.573
Pro-democracy	195	0.398	0.490	199	0.400	0.490	0.938
Others	94	0.192	0.394	96	0.193	0.395	0.958
Attention to Coronavirus	484	2.366	1.075	491	2.432	1.069	0.336
Knowledge about Coronavirus	490	0.512	0.500	497	1.127	0.993	0.107
Perceived Risk of Being Infected	490	2.080	0.819	497	2.034	0.841	0.391
Perceived China's Influence on Hong Kong	443	2.077	1.267	452	2.046	1.253	0.719
Participated in the 2019 Protests	462	0.305	0.461	458	0.317	0.466	0.709
Support for Media Freedom	488	2.523	0.808	494	2.496	0.815	0.608

Note: The p-values of two-tailed t-tests are reported in the last column.

S2 Table. Randomization checks: Experiment 2

Variable	Without Gov. Title			With Gov. Title			p-value
	N	Mean	SD	N	Mean	SD	
Age	484	43.262	12.936	503	43.897	13.373	0.449
Male	484	0.519	0.500	506	0.457	0.499	0.051
College	481	0.669	0.471	505	0.648	0.478	0.469
Subjective Social Class	468	2.323	0.841	491	2.248	0.885	0.489
Party ID	484			506			
Pro-establishment	61	0.126	0.332	68	0.134	0.341	0.697
Moderate	134	0.277	0.448	142	0.281	0.450	0.895
Pro-democracy	192	0.397	0.490	201	0.397	0.490	0.986
Others	97	0.200	0.401	95	0.188	0.391	0.615
Attention to Coronavirus	476	2.408	1.083	502	2.384	1.056	0.736
Knowledge about Coronavirus	484	1.103	0.996	506	1.055	0.999	0.450
Perceived Risk of Being Infected	484	2.056	0.826	506	2.055	0.833	0.993
Perceived China's Influence on Hong Kong	434	2.018	1.208	464	2.097	1.295	0.348
Participated in the 2019 Protests	462	0.307	0.462	461	0.317	0.466	0.760
Support for Media Freedom	482	2.529	0.800	503	2.497	0.818	0.535
Heard of Chen	484	0.599	0.491	506	0.625	0.484	0.414
Independence of Chen	290	1.997	0.732	316	2.016	0.706	0.741

Note: The p-values of two-tailed t-tests are reported in the last column.

Supplementary information D: Operationalization of variables

S3 Table. Operationalization of variables

Variable	Question Wording and Operationalization
Male	1: Male; 0: Female
College	1: With a College; 0: Without a college degree
Subjective Social Class	"In your view, which social class does your family belong to?"<br>1: Working or Lower class; 2: Lower middle class; 3: Middle class;<br>4: Upper middle class, 5: Upper class. DKs are dropped.
Partisanship	"What's your political leaning?" 1: Pro-establishment group;<br>2. Moderate group; 3: Pro-democracy group; 4: Others (including DKs)
Attention to Coronavirus	"In the past week, how much time did you spend daily on learning<br>information about the outbreak of NCP."<br>1: Not interested at all or less than one hour; 2: 1-2 hours;<br>3: 2-3 hours; 4: More than 3 hours; DKs are dropped.
Knowledge about<br>Coronavirus	"Do you agree with the following statements? "<br>'Only N-95 masks can prevent the infection of NCP.'<br>'In addition to bleach and ethanol, vinegar can also kill the<br>virus to prevent the spread of NCP.'<br>0: If respondents answer "Yes" to both questions;<br>1: If respondents answer only one "No" to either questions;<br>2: If respondents answer "No" to both questions.
Perceived Risk of<br>Being Infected	"How likely do you think it is that you will get infected by the NCP?"<br>1: Not possible at all or somewhat unlikely<br>2: DK; 3: Somewhat likely or very likely.
Perceived China's Influence<br>on Hong Kong	"Overall, what's your opinion about mainland China's impact<br>on Hong Kong?" 1: Very negative; 2: Somewhat negative;<br>3: No impact; 4: Somewhat positive; 5: Very positive. DKs are dropped.
Participated in the 2019<br>Protests	"Have you participated in the Anti-Extradition<br>Protests in any form?" 0: No; 1: Yes. DKs are dropped.
Support for Media Freedom	"Which of the following two statements best reflects your opinion?"<br>1: Government has the right to refrain the media from reporting the<br>news that may result in political instability; 2: DK;<br>3: Media have the right to report news and express opinions,<br>free from the government control.
Heard of CQ	"Have you heard about a citizen reporter named Chen Qiushi?"<br>0: No; 1: Yes.
Independence of CQ	"Do you think Chen Qiushi's report is independent and not under<br>the government's instruction?" 0: No; 1: Don't Know; 2: Yes.
Perceived Government<br>Transparency	"Overall, what is the level of transparency about the NCP outbreak<br>in different levels of government (Central/Wuhan/Hong Kong)?" 1: Very low;<br>2: Somewhat Low; 3: Somewhat high; 4: Very high. DKs are dropped.
Trust in Government	"In your opinion, how trustworthy is PRC's Central/Wuhan/Hong Kong<br>government?" 1: Very untrustworthy; 2: Somewhat untrustworthy;<br>3: Somewhat trustworthy; 4: Very trustworthy. DKs are dropped.

Note: DK = Don't know; NCP: Novel Coronavirus Pneumonia.

Supplementary information E: Summary Statistics

S4 Table. Summary statistics

Variable	N	Mean	SD	Minimum	Maximum
Trust in					
Government's Press Release	987	44.424	27.527	0	100
Report of the People's Daily	490	43.753	28.192	0	100
Report of Chen Qiushi	491	53.259	25.278	0	100
Medical Expert without Government Title	506	49.753	27.609	0	100
Medical Expert with Government Title	484	49.787	27.609	0	100
Age	1,013	43.358	13.142	18	80
Male	1,016	0.483	0.500	0	1
College	1,007	0.659	0.474	0	1
Subjective Social Class	978	2.289	0.864	1	5
Party ID	1,016				
Pro-establishment	61	0.128	0.334	0	1
Moderate	140	0.272	0.445	0	1
Pro-democracy	195	0.401	0.490	0	1
Others	94	0.200	0.400	0	1
Attention to Coronavirus	1,004	2.404	1.070	1	4
Knowledge about Coronavirus	1,016	1.085	0.997	0	2
Perceived Risk of Being Infected	1,016	2.063	0.828	1	3
Perceived China's Influence	922	2.051	1.253	1	5
Participated in the 2019 Protests	939	0.315	0.465	0	1
Support for Media Freedom	1,009	2.519	0.805	1	3
Heard of Chen	1,016	0.611	0.488	0	1
Independence of Chen	621	2.008	0.715	1	3

Supplementary information F: Results of OLS Models for Experiment 1

This section describes how we used various OLS regression models to investigate the endorsement effects of a citizen journalist, Chen Qiushi, on the press release of the Chinese government. We included variables on respondents' demographic traits and attitudes, such as their age, gender, education, subjective social class, attitudes toward the coronavirus, perception of China's influence on Hong Kong, participation in the 2019 Anti-Extradition protests in Hong Kong, support for media media freedom, awareness of Chen, and perception of his independence from the government. We also investigated whether the endorsement effect is heterogeneous among respondents by including interaction terms between their attitudes and the treatment assignment (Models 2 to 5). We illustrated the heterogeneous effects by drawing marginal effects plots.

Model 1 suggests that the group of respondents who read Chen's report would have more trust of 4.10 points on a scale from 0 to 100 in the government's press release than those who read the report of People's Daily (p = 0.008, 95% CI 1.06-7.14). Model 2 shows that respondents' skepticism of the Chinese government would be mitigated after reading Chen's report. The coefficient of "Participated in the 2019 Protests" is negative (-10.85, $p < 0.001$, 95% CI -15.83-5.88), and its interaction term with Group Chen is positive (5.75, p = 0.075, 95% CI -0.59-12.08). Meanwhile, difference in the effect between reading People's Daily and Chen's report is indistinguishable for the respondents who attended and did not attend the protests.

We also investigated whether there was a heterogeneous effect among those who support the media freedom or its suppression by the government in China. The respondents who supported media freedom may be skeptical of government's press release because China had limited freedom of media and People's Daily was the Chinese government's mouthpiece. In contrast, those who supported government suppression of media freedom would be less skeptical of the People's Daily and give higher ratings to the government's credibility. The results of Model 3, as illustrated in Figure 4 in the main text, suggest that respondents who supported media freedom would trust the government's news by 6 points more if they read Chen's report instead of the People's Daily (45.17, $p < 0.001$, 95% CI 42.56-47.78 versus 39.60,$p < 0.001$, 95% CI 37.07-42.13). Meanwhile, the difference in the effect between reading People's Daily and Chen's report is indistinguishable for the respondents who supported government suppression of media freedom in China or did not have a clear position on media suppression versus freedom. In other words, the respondents who supported media freedom would be more skeptical of the government's press release, but they would also upwardly adjust their trust in the government policy release significantly more if they learned that that the press release was endorsed by a non-official source of information.

Models 4 and 5 further explore whether respondents' knowledge of Chen makes his report more influential. In Model 4, we included a dummy variable to indicate whether respondents' had heard of Chen, and we also interacted that variable with Group Chen. Figure 4 in the main text illustrates the marginal effect of reading Chen's report and shows that the respondents who have heard of Chen before trusted the official press release more by 6.44 points than those who supported media freedom but read the People's Daily report (48.34, $p < 0.001$, 95% CI 45.52-51.16 versus 41.90, $p < 0.001$, 95% CI 39.09-44.72). Meanwhile, it should be noted that if respondents had never heard of Chen before, the difference in marginal effect between reading Chen's and the People Daily's report is statistically indistinguishable (42.54, $p < 0.001$, 95% CI 39.05-46.04 versus 42.61, $p < 0.001$, 95% CI 39.13-46.09). Thus, the endorsement effect of Chen is much higher among those respondents who have heard of him.

In Model 5, we explored whether it is Chen's image as an independent citizen

reporter that makes him more trustworthy. We included a variable that measured respondents' perception of Chen's independence from the government. We interacted this variable with Group Chen, with the baseline group as respondents who read the People's Daily. Figure 4 in the main text shows that when respondents believed that Chen was an independent reporter, they trusted the government's press release more after they read Chen's endorsement of it by 9.46 points (46.87, $p < 0.001$, 95% CI 42.11-51.63 versus 37.41, $p < 0.001$, 95% CI 32.77-42.06). Yet, the respondents who regarded Chen as not independent trusted the government's press release less after they read his report (36.95, $p < 0.001$, 95% CI 32.04-41.86 versus 33.84, $p < 0.001$, 95% CI 29.14-38.53). Meanwhile, those who did not know Chen's independence would have a middle level of trust in the press release (35.64, $p < 0.001$, 95% CI 32.83-38.42 versus 41.91, $p < 0.001$, 95% CI 39.16-44.66). Thus, the endorsement effect of Chen on the trustworthiness of the official news results from respondents' perceived level of Chen as an independent reporter.

S5 Table. Determinants of popular trust in the Ministry of Commerce's press release

	Model 1	Model 2	Model 3	Model 4	Model 5
Group Chen	4.097**	2.129	-3.899	-0.066	-0.058
	[1.549]	[1.901]	[4.662]	[2.393]	[5.953]
Age	0.152*	0.147*	0.156*	0.154*	0.140
	[0.066]	[0.066]	[0.066]	[0.066]	[0.089]
Male	2.011	1.953	1.979	1.818	0.598
	[1.599]	[1.599]	[1.598]	[1.596]	[2.063]
College	-2.057	-2.071	-2.031	-1.975	-1.128
	[1.888]	[1.887]	[1.882]	[1.891]	[2.577]
Subjective Social Class	1.652	1.746+	1.727	1.635	1.431
	[1.057]	[1.052]	[1.052]	[1.055]	[1.399]
Partisanship: Moderate	-7.821**	-7.793**	-7.890***	-8.103***	-7.246+
	[2.395]	[2.389]	[2.386]	[2.394]	[3.724]
Partisanship: Pro-democracy	-11.617***	-11.558***	-11.918***	-12.220***	-12.745**
	[2.726]	[2.713]	[2.721]	[2.725]	[4.002]
Partisanship: Others	-9.008**	-8.981**	-9.346**	-9.300**	-12.541**
	[2.908]	[2.894]	[2.902]	[2.907]	[4.703]
Attention to Coronavirus	-0.184	-0.145	-0.237	-0.295	0.727
	[0.750]	[0.748]	[0.749]	[0.751]	[1.010]
Knowledge about Coronavirus	0.216	0.257	0.216	0.140	0.054
	[0.778]	[0.777]	[0.777]	[0.777]	[1.004]
Perceived Risk of Being Infected	-1.494	-1.500	-1.445	-1.520	-2.144+
	[0.935]	[0.934]	[0.933]	[0.938]	[1.216]
Perceived China's Influence	7.526***	7.568***	7.510***	7.607***	8.013***
on Hong Kong	[0.831]	[0.823]	[0.827]	[0.831]	[1.150]
Participated in the 2019 Protests	-7.924***	-10.854***	-7.826***	-8.374***	-9.216***
	[1.987]	[2.532]	[1.986]	[1.998]	[2.409]
Support for Media Freedom	-4.002***	-3.999***	-5.572***	-4.334***	-4.554*
	[1.102]	[1.099]	[1.444]	[1.114]	[1.799]
Group Chen X		5.747+			
Participated in the 2019 Protests		[3.227]			
Group Chen X			3.156+		
Support for Media Freedom			[1.800]		
Heard of Chen				-0.708	
				[2.337]	
Group Chen X				6.507*	
Heard of Chen				[3.121]	
Independence of Chen					1.787
					[1.905]
Group Chen X					3.170
Independence of Chen					[2.783]
Constant	40.937***	41.724***	44.802***	42.968***	39.303***
	[6.381]	[6.396]	[6.792]	[6.575]	[9.792]
R-squared	0.402	0.405	0.404	0.407	0.368
N	806	806	806	806	513

[flushleft]

Note: Robust standard errors in brackets. The marginal effects of interaction terms in Models 2 to 5 are illustrated in Figures 3 to 4 in the main text. + $p < 0.1$, * $p < 0.05$, ** $p < 0.01$, *** $p < 0.001$. All tests are two-tailed.

Supplementary information G: Age and Heterogeneous Treatment Effects

Given the generational gap in Hong Kong's political landscape, age as a factor might play a significant role in moderating the effect of our treatment on the outcome variable. In the section we conduct an additional analysis by including an interaction between age and the treatment in the model. While the coefficient of age in this interaction model is 0.185 with a p-value of 0.025, the coefficient of the interaction term between age and the treatment is -0.064 with a p-value of 0.558. Nevertheless, since what we focus on here is the difference in treatment effects across respondents' age, we present in S3 Fig Fig below the marginal effect of treatment across the range of age in our sample (18-80). As the figure shows, the difference in treatment effects decreases from 5.23 for 18-year-old respondents to 1.77 for 80-year-old ones. Meanwhile, the differences in marginal treatment effect are statistically significant at the $p < 0.05$ level for respondents aged between 24 and 52 years old. In other words, the treatment effect is larger for younger respondents than their senior counterparts.

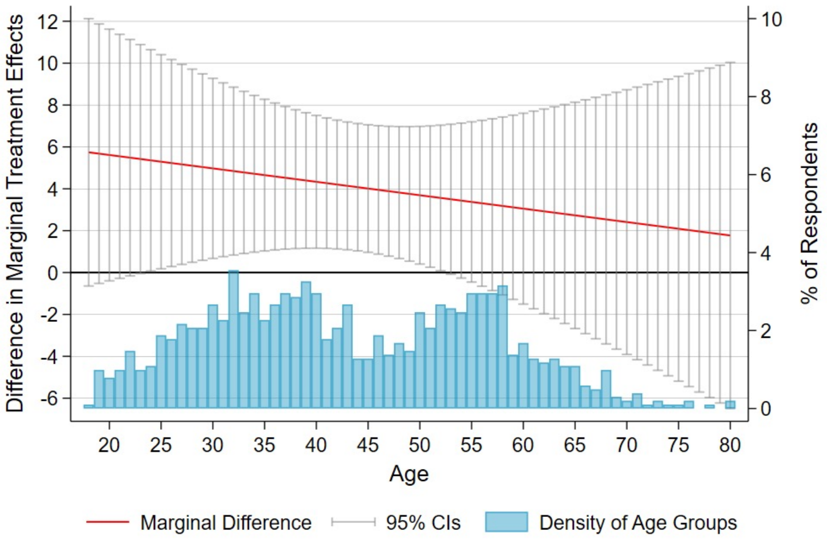

S3 Fig. Age and Heterogeneous Treatment Effects.

Supplementary information H: The Questionnaire (in English)

1. Thank you for participating in this research! This is a survey on the news coverage of 2019 Novel Coronavirus Pneumonia. It will take you about 10-15 minutes to complete the survey. Before starting the survey, please read and sign the informed consent form below. Participating in this study is entirely voluntary, and you can stop answering the survey at any time. Please click here to download: Informed consent form. Do you agree to participate in this study? [Agree; Disagree]

2. Research shows that individual decision-making is affected by many factors, such as personal preferences, educational background, knowledge, and other environmental factors. To ensure the validity of this study, we want to understand what factors affect your decision-making. We also want to ensure each respondent has read the instructions in the survey carefully in order to increase the overall reliability of the survey. Whether you use email or not, please choose "yes" to the statement below, and then proceed. I have never used email. [Yes; No; I don't know]

3. Research shows that 75% ethanol can be used for cleaning and antiseptic purpose.s Once again, we want to ensure each respondent has read the instructions in the survey carefully in order to increase the overall reliability of the survey. Please move the slider below to 75, and then proceed.

4. What is your gender? [Male; Female]

5. What is your birth year?

6. In the past week, how much time did you spend daily in learning information about the outbreak of Novel Coronavirus Pneumonia? [Not interested at all; Less than one hour; 1-2 hours; 2-3 hours; More than 3 hours; I don't know]

7. Where do you acquire the information about Novel Coronavirus Pneumonia? (Choose all that apply) [Hong Kong mass media (including TV, newspaper, radio, and magazine); Mainland China mass media (including TV, newspaper, radio, and magazine); Foreign mass media (including TV, newspaper, radio, and magazine); Hong Kong social media; Mainland China social media; Foreign social media; Internet; Family members or friends; Other]

8. If information from different sources is contradictory, which information source do you trust the most? [Hong Kong mass media (including TV, newspaper, radio, and magazine); Mainland China mass media (including TV, newspaper, radio, and magazine); Foreign mass media (including TV, newspaper, radio, and magazine); Hong Kong social media; Mainland China social media; Foreign social media; Internet; Family members or friends; Other]

9. What is your perceived likelihood of being infected with Novel Coronavirus Pneumonia? [Not possible at all; Unlikely; Somewhat likely; Very likely; I don't know]

10. No one wants to be infected with Novel Coronavirus Pneumonia, but if you are unfortunately infected, are you confident that you will be able to access to adequate medical care? [Very confident; Confident; Somewhat not confident; Not confident at all; I don't know]

11. Do you have any relative or friend who has been infected with Novel Coronavirus Pneumonia? [Yes; No; I don't know]

12. Do you agree with the following statement? "Only N-95 masks can prevent the infection of Novel Coronavirus Pneumonia." [Agree; Disagree; I don't know]

13. Do you agree with the following statement? "In addition to bleach and ethanol, vinegar can also kill the virus to prevent the spread of Novel Coronavirus Pneumonia." [Agree; Disagree; I don't know]

14. After the outbreak of Novel Coronavirus Pneumonia, have you washed your hands more? [Not at all; Some; A lot; Extremely; I don't know]

15. How often do you acquire information about politics and government from the Internet and social media? [Almost daily; More than twice in a week; A couple of times in a week; Several times in a month; Almost never; I don't know]

16. Which of the following two statements about the relationship between the government and the media best reflects your opinion? [The Media have the right to report news and express opinions, free from government control; The government has the right to refrain the media from reporting news that may result in political instability;I don't know]

17. Have you heard about a citizen reporter named Chen Qiushi? [Yes, I have heard of him; No, I have never heard of him before]

18. Do you know the occupation of Chen Qiushi? [Lawyer; Engineer; Banker; Medical doctor; I don't know]

19. Do you think Chen Qiushi's report is objective and unbiased? [Yes; No; I don't know]

20. Do you think Chen Qiushi's report is independent and not under the government's instructions? [Yes; No; I don't know]

21. Overall, what's your opinion about mainland China's impact on Hong Kong? [Very positive; Positive; Somewhat positive; Somewhat negative; Negative; Very negative; No impact; I don't know]

22. Have you participated in the Anti-Extradition Law Amendment Bill Movement in any form? [Yes; No; I don't know]

23. What was the nature of your participation? (Choose all that apply) [Joined peaceful parades; Showed support for the movement online; Other]

24. After the outbreak of Novel Coronavirus Pneumonia, do you wear a mask when visiting public places? [Never; Sometimes; Usually; Always]

25. What type of masks do you wear? [N-95 mask; Surgical mask; Charcoal mask; Cotton mask; I don't know]

26. After the outbreak of Novel Coronavirus Pneumonia, what do you observe about people wearing masks in public places? [Not at all; More; Less; Almost everyone; I don't know]

27. Have you decreased the frequency of the following activities after the outbreak of Novel Coronavirus Pneumonia?

    (a) Being in public space [Not at all; A little; A lot; Not sure]

(b) Using public transportation [Not at all; A little; A lot; Not sure]

28. (Experiment 1: Treatment Group) [Ministry of Commerce press release+ Chen Qiushi's endorsement] The day after the Ministry of Commerce made the statement, citizen reporter Chen Qiushi visited a supermarket in Wuhan and made a report. Below is a screenshot from his video.

    (a) Do you believe in the Ministry of Commerce's press release? Rate it on a scale from 0-100. A larger number indicates a higher level of trust.

    (b) Do you believe that the report by Chen Qiushi faithfully reflected the situation in Wuhan? Rate it on a scale from 0-100. A larger number indicates a higher level of trust.

29. (Experiment 1: Control Group) [Ministry of Commerce press release+ People's Daily's endorsement]

    (a) Do you believe in the Ministry of Commerce's press release? Rate it on a scale from 0-100. A larger number indicates a higher level of trust.

    (b) Do you believe that the report by Chen Qiushi faithfully reflected the situation in Wuhan? Rate it on a scale from 0-100. A larger number indicates a higher level of trust.

30. (Vignette mentioning the expert's government-related title) Do you believe in the opinion of Dr. Li Xingwang, a member of National Medical Expert Committee and Chief Expert at Clinical and Research Center of Infectious Diseases, Beijing Ditan Hospital? Rate it on a scale from 0-100. A larger number indicates a higher level of trust.

31. Overall, what do you think about the levels of information transparency of different governments in the outbreak of Novel Coronavirus Pneumonia?

    (a) The central government of PRC [Very low; Somewhat low; Somewhat high; Very high;I don't know]

    (b) Hong Kong government [Very low; Somewhat low; Somewhat high; Very high;I don't know]

    (c) Wuhan city government [Very low; Somewhat low; Somewhat high; Very high;I don't know]

32. Do you think the statistics about Novel Coronavirus Pneumonia announced daily by the National Health Commission are precise, under-, or overestimated? [Highly overestimated; Overestimated; Precise; Underestimated; Highly underestimated; I don't know]

33. What are your levels of trust in the following institutions and groups?

    (a) Mainland Chinese "Media"

    (b) The central government of PRC [Highly trustworthy; Somewhat trustworthy; Somewhat untrustworthy; Very untrustworthy; I don't know]

    (c) Hong Kong government [Highly trustworthy; Somewhat trustworthy; Somewhat untrustworthy; Very untrustworthy; I don't know]

    (d) Mainlanders [Highly trustworthy; Somewhat trustworthy; Somewhat untrustworthy; Very untrustworthy; I don't know]

    (e) Hong Kongers [Highly trustworthy; Somewhat trustworthy; Somewhat untrustworthy; Very untrustworthy; I don't know]

(f) Mainland Chinese ”Citizen Journalists, Streamers” [Highly trustworthy; Somewhat trustworthy; Somewhat untrustworthy; Very untrustworthy; I don’t know]

(g) Mainland Chinese ”Local Governments” [Highly trustworthy; Somewhat trustworthy; Somewhat untrustworthy; Very untrustworthy; I don’t know]

34. Do you support the Hong Kong government donating medical supplies such as facial masks to mainland China? [Very supportive; Somewhat supportive; Not so supportive; Not supportive at all I don’t know]

35. Overall, are you satisfied with how the different governments addressed the outbreak of Novel Coronavirus Pneumonia?

    (a) The central government of PRC [Very satisfied; Satisfied; Not satisfied; Very not satisfied; I don’t know]

    (b) Hong Kong government [Very satisfied; Satisfied; Not satisfied; Very not satisfied; I don’t know]

    (c) Wuhan city government [Very satisfied; Satisfied; Not satisfied; Very not satisfied; I don’t know]

36. Do you think the level of freedom of speech in mainland China should increase? [Very in need; In need; Not in need; Very not in need; I don’t know]

37. Do you support the medical workers’strike in Hong Kong during the outbreak of Novel Coronavirus Pneumonia? [Supportive; Not supportive; I don’t know]

38. Do you agree with the following statement: ”Citizen journalists and streamers help me understand more about Novel Coronavirus Pneumonia” [Very agree; Agree; Not agree; Very not agree; I don’t know]

39. Have you been to mainland China in the past half year? [Yes; No; Prefer not to say]

40. What is your highest level of education? [No formal education; Elementary school; Junior high school; Senior high school; College or junior college; Graduate school or above]

41. What social class do you consider your family to belong to? [Upper class; Upper middle class; Middle class; Lower middle class; Working or lower class; I don’t know]

42. What’s your occupation? [Professional; Service; Labor; Student; Homemaker; Retired; Between jobs; Other]

43. What is your political leaning? [Pro-democracy group; Pro-establishment group; Moderate group; Other]

44. Where were you born? [Hong Kong; Mainland China; Taiwan; Macao; Southeast Asia countries Canada; United States of America; Australia; United Kingdom; Others]

45. Do you have relatives living in mainland China? [Yes; No; I don’t know]

46. Do you have business or investments in mainland China? [Yes; No; Not now but used to have; I don’t know]

47. What is your place of residence?
